# Qualitative and Quantitative Assessment of Vitreous Inflammation in Uveitis: Current Limitations and Emerging Diagnostic Approaches

**DOI:** 10.3390/diagnostics16121886

**Published:** 2026-06-17

**Authors:** Maria Carmela Saturno, Oscar Matteo Gagliardi, Maurizio La Cava, Chiara Ciccarè, Alice Bruscolini, Alessandro Lambiase, Danilo Iannetta

**Affiliations:** Department of Sense Organs, Sapienza University of Rome, 00161 Rome, Italy; oscarmatteo.gagliardi@uniroma1.it (O.M.G.); maurizio.lacava@uniroma1.it (M.L.C.); chiara.ciccare@uniroma1.it (C.C.); alice.bruscolini@uniroma1.it (A.B.); alessandro.lambiase@uniroma1.it (A.L.); danilo.iannetta@uniroma1.it (D.I.)

**Keywords:** uveitis, vitritis, vitreous inflammation, optical coherence tomography, quantitative imaging, biomarkers, artificial intelligence

## Abstract

Accurate assessment of vitreous inflammation is essential for the diagnosis, monitoring and management of uveitis. Traditionally, vitritis has been evaluated using subjective clinical grading systems based on vitreous haze and cellular infiltration, which are limited by interobserver variability and poor reproducibility, particularly in cases of mild or subclinical inflammation. In recent years, advances in ocular imaging have enabled the development of more objective, quantitative approaches. Ultra-widefield imaging, optical coherence tomography (OCT) and ultrasound-based techniques have provided new insights into structural alterations within the vitreous. In parallel, automated image analysis and artificial intelligence (AI)-based methods have improved the detection and quantification of inflammatory biomarkers, including vitreous hyperreflective foci and signal intensity-based metrics. Despite these advances, important limitations remain, including a restricted field of view, a lack of standardized segmentation algorithms and an incomplete representation of the entire vitreous cavity. No single modality currently provides a comprehensive and fully reproducible assessment of vitreous inflammation. This review summarizes current qualitative and quantitative methods for evaluating vitreous inflammation, highlighting their respective strengths and limitations. In addition, emerging diagnostic strategies, including multimodal imaging integration, AI-driven analysis and molecular biomarker profiling, are discussed as potential tools to improve accuracy, standardization and clinical applicability. The transition from subjective grading toward objective quantification of inflammatory burden represents a key step in advancing both clinical management and research in ocular inflammatory diseases.

## 1. Introduction

The vitreous body occupies a volume of about 4.5 mL and is the largest single structure in the eye, accounting for approximately 80% of its total volume. Anteriorly, it is delineated by and adjoins the ciliary body, zonules and lens, while posteriorly it lies adjacent to the retina. It is a transparent, gel-like, optically clear substance composed predominantly of water (98–99%), with a network of collagen fibrils, hyaluronic acid and various proteoglycans. This composition helps preserve the shape of the eye, supports the retina and maintains a transparent medium for light transmission [1,2].

Nevertheless, inflammatory and/or infectious processes may alter the structural components of the vitreous. Immune cell infiltration, including macrophages and lymphocytes, together with the accumulation of proteinaceous exudates and cellular debris, leads to the formation of vitreous opacities and aggregates [1,3,4,5]. These changes disrupt vitreous transparency and contribute to the characteristic features observed in ocular inflammatory disorders, representing a clinical hallmark of vitritis [1,4,5,6].

Vitritis is a prominent clinical manifestation of intermediate, posterior and panuveitis. If not properly managed, the accumulation of inflammatory cells and opacities may result in visual impairment [1,4,6]. Accurate and timely evaluation is therefore essential for diagnosis, monitoring disease progression and assessing therapeutic response [1,4,5,6].

In clinical practice, vitreous inflammation is traditionally assessed using slit-lamp biomicroscopy and indirect ophthalmoscopy. The National Eye Institute (NEI) vitreous haze scale, later standardized by the Standardization of Uveitis Nomenclature (SUN) workshop in 2005, remains the most widely used clinical grading system for vitreous inflammation [7]. However, these approaches rely on subjective interpretation and show limited sensitivity, particularly in cases of mild or subclinical disease [6]. Advances in ocular imaging, including optical coherence tomography (OCT), OCT angiography (OCTA) and ultrasound, have improved detection by providing detailed structural information [5,6,8]. Nevertheless, these techniques still present important limitations, particularly regarding the ability to capture the full extent of inflammatory changes within the vitreous cavity [1,6,9].

A systematic review of clinical trials in intermediate, posterior and panuveitis identified substantial heterogeneity in outcome measures, with 14 different domains and vitreous haze used in 57% of disease activity assessments, highlighting the lack of consensus and the need for standardized and reproducible evaluation methods [10].

In this context, there is an increasing need for objective, reproducible and quantitative assessment methods. This narrative review summarizes current qualitative and quantitative approaches for vitreous inflammation assessment, focusing on clinical grading systems, multimodal imaging, artificial intelligence and emerging biomarkers.

### 1.1. Clinical Methods for Quantitative Assessment of Vitreous Inflammation

Historically, vitreous inflammation has been assessed through clinical examination, with ophthalmologists estimating cellular infiltration, vitreous opacities and haze based on clinical experience [3,6,7]. Vitreous haze reflects diffuse turbidity of the vitreous and is typically evaluated indirectly through the degree of obscuration of fundus details [1,3,6]. In addition, inflammatory exudates may produce vitreous flare, best described by the Tyndall effect, which refers to the scattering of light by particles suspended within the vitreous cavity [1,3].

Early attempts to standardize clinical evaluation date back to 1959, when the Protocol Group at the University of California, San Francisco, proposed one of the first classification systems for vitreous inflammation. In this work, retrolental inflammatory cells were assessed using slit-lamp retroillumination with a Hruby lens, and both vitreous cells and flare were graded on a scale from 0+ to 4+. Vitreous opacities were categorized as fine, coarse, stringy and snowball formations, with snowballs typically located in the inferior vitreous base and ora serrata and potentially coalescing into snowbanks in more advanced cases [3].

To improve standardization, the Nussenblatt grading system was introduced in 1985 as a structured clinical tool for assessing vitreous haze in intermediate and posterior uveitis. This photographic-based scale compared the degree of vitreous haze in patients’ eyes with standardized reference images, grading inflammation from 0+ (no haze) to 4+ (severe haze) [11]. Subsequently, the SUN workshop in 2005 introduced a six-step grading scale for vitreous haze, including a 0.5+ category to improve sensitivity for mild inflammation and to standardize reporting across clinical studies [7].

Despite these advances, limitations persist, particularly in assessing low-grade inflammation and subtle changes over time. Interobserver variability continues to affect both cross-sectional and longitudinal evaluation, potentially influencing clinical decision-making and treatment strategies. To address these issues, alternative grading systems have been proposed. Among these, the Miami scale, a 9-point photographic grading system developed by Davis et al., allows for finer discrimination of vitreous haze severity and improves sensitivity for detecting mild inflammatory changes. This scale demonstrated high reliability, with intraclass correlation coefficients ranging from 0.84 to 0.91 and κ values averaging 0.91, indicating near-perfect agreement, while 87.7% of grading variability was attributable to the test item rather than observer-related factors, supporting its robustness and suitability for clinical trials [12].

In parallel, advances in imaging technologies have enabled widefield (WF) and ultra-widefield (UWF) fundus imaging. These modalities have demonstrated strong interobserver reliability (κ ≥ 0.886) and good correlation with traditional clinical grading systems, suggesting that imaging-based metrics may provide a more reproducible assessment of vitritis severity [13,14]. Furthermore, the wider retinal field captured by UWF imaging may facilitate documentation of disease extent and longitudinal monitoring of inflammatory changes in posterior uveitis. This feature may be particularly valuable for standardized follow-up and objective assessment of treatment response. However, limitations remain; for example, Dickson et al. reported that ultra-widefield scanning laser ophthalmoscopy (UWF-SLO) may be less sensitive than conventional fundus photography in detecting mild vitreous haze [15].

### 1.2. Clinical Methods for Qualitative Assessment of Vitreous Inflammation and Disease-Specific Pattern

As for qualitative clinical features, they continue to play a significant diagnostic role. Specific patterns of vitreous opacities and cellular distribution may provide clues to the underlying disease. Pars planitis, for example, is characterized by inferior snowball opacities associated with peripheral retinal periphlebitis and snowbank formation in the absence of systemic disease [13]. Large confluent snowball opacities in ocular sarcoidosis may produce the characteristic “string of pearls” appearance [16]. In Behçet disease, vitreous inflammation commonly occurs in association with retinal vasculitis and focal retinal infiltrates [17]. In ocular toxoplasmosis, vitritis is an important component of the host immune response and typically accompanies a focal necrotizing retinitis, often presenting as an active round or oval lesion adjacent to a pigmented retinochoroidal scar [18]. In primary vitreoretinal lymphoma, vitreous involvement is typically characterized by dense cellular infiltration forming sheets or clumps associated with subretinal infiltrates [19]. Despite their diagnostic relevance, these findings remain largely subjective and typically require integration with laboratory and imaging investigations to establish a definitive diagnosis.

### 1.3. Imaging Qualitative Assessment of Vitreous Inflammation

Imaging-based assessment of vitreous inflammation has been increasingly developed to provide more objective and reproducible methods compared with clinical evaluation. Among the available modalities, OCT [1] and Dynamic Infrared Imaging (DIR) [20] are two tools for the qualitative assessment of vitreous changes.

OCT is a non-invasive imaging technique based on the reflection of light waves from ocular structures such as the retina and vitreous body, enabling high-resolution cross-sectional visualization at a micrometric scale [1,6,8]. While OCT has become indispensable for evaluating macular diseases, its role in qualitative assessment of vitreous inflammation remains limited. Pichi et al. explored the use of en-face OCT to visualize posterior vitreous anatomy in patients with anterior uveitis, demonstrating the presence of optically empty spaces and suggesting a possible connection between the premacula bursa and Cloquet’s canal, potentially facilitating the diffusion of inflammatory cytokines involved in the development of cystoid macular edema in these patients [21]. Despite these insights, OCT provides only partial visualization of vitreous alterations. Its principal limitation is the restricted field of view, which prevents a comprehensive evaluation of the entire vitreous cavity.

DIR is an emerging imaging technique that uses a focused laser beam to scan the retina, acquiring two-dimensional sections through oscillating mirrors. Using wavelengths typically around 820 nm, DIR generates infrared reflectance images in which variations in light intensity are detected with high sensitivity. This allows for the visualization of vitreous opacities, which appear as areas of light obscuration due to increased vitreous density and enables dynamic assessment of their movement during saccades [20]. Although DIR has yet to be systematically evaluated in vitritis, it may represent a promising qualitative modality for detecting inflammatory changes within the vitreous. Nevertheless, similar to OCT, its application is limited by a relatively narrow field of view, approximately 30 degrees, which allows for detailed visualization of the macular region but does not permit a comprehensive assessment of the entire vitreous cavity.

### 1.4. Imaging Quantitative Assessment of Vitreous Inflammation

Quantitative imaging approaches for vitritis have been developed in recent years to overcome the limitations of subjective clinical grading and to improve the detection of subtle inflammatory changes. Among these, OCT has emerged as a key modality, demonstrating efficacy in identifying both vitreous cells and diffuse inflammatory alterations [1,22].

**Figure 1:**
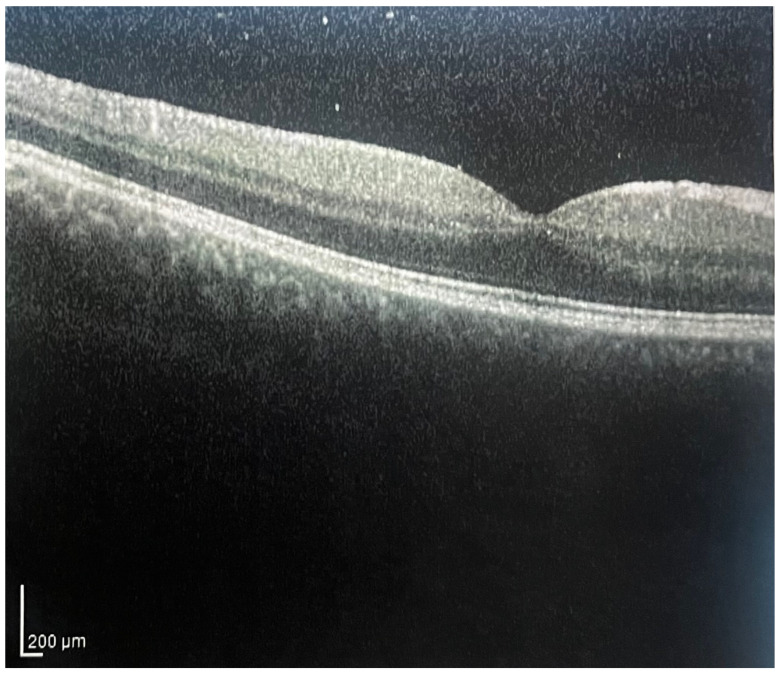
Optical coherence tomography (OCT) image showing multiple hyperreflective foci within the posterior vitreous cavity, consistent with inflammatory cells. These punctate elements represent vitreous cellular infiltration and can serve as biomarkers of disease activity. Image obtained from the authors’ institutional database after written informed consent.

Masaaki et al. conducted a pilot study using swept-source OCT (SS-OCT) to visualize posterior vitreous cells in patients with intraocular inflammation. Hyperreflective elements within the posterior vitreous were manually counted, revealing significantly higher numbers in eyes with active disease [23] (Figure 1). Similarly, Llorenç et al. reported an increased prevalence of vitreous reflective signals in inflamed eyes [24]. However, distinguishing discrete cellular aggregates from diffuse vitreous reflectivity remains challenging, limiting accurate quantification and highlighting the need for improved segmentation techniques and standardized image analysis.

To address these limitations, Keane et al. introduced the VITAN (VITreous ANalysis) system in 2015 [25], representing a major step toward automated OCT-based quantification. This method applies advanced algorithms to detect and quantify clusters of high-intensity pixels corresponding to aggregates of inflammatory cells. By analyzing parameters such as cluster number, size and maximal dimensions, the system provides objective quantitative output. Importantly, automated texture analysis showed significant correlation with clinical vitreous haze grading (r = 0.604, *p* < 0.001), supporting its potential as a reproducible, observer-independent measure of intraocular inflammation [25].

Building on this concept, Lee et al. employed spectral-domain OCT (SD-OCT) combined with a Python-based analysis script to quantify vitreous reflective foci in uveitis, including their number, size and total area. Longitudinal evaluation demonstrated a reduction in these parameters following treatment, suggesting their potential as dynamic biomarkers of disease activity and therapeutic response [26]. Similarly, Korot et al. applied automated quantification of vitreous reflectivity in diabetic retinopathy, demonstrating correlation with the severity of diabetic macular edema and supporting their role as non-invasive markers of inflammatory burden and disease progression [27].

In the context of differential diagnosis, Köksaldı et al. used ImageJ analysis software to demonstrate that vitreous reflective foci observed in acute toxoplasma chorioretinitis were significantly larger compared to those in non-infectious uveitis. However, the biological nature of vitreous hyperreflective foci remains incompletely understood. These structures are generally believed to reflect inflammatory cellular infiltrates and may represent inflammatory cells migrating into the vitreous. However, structural OCT alone cannot determine their precise cellular composition. Therefore, caution is warranted when interpreting hyperreflective foci as direct markers of inflammatory cell burden [28].

Beyond cellular aggregates, signal intensity in the vitreous may serve as an additional quantitative parameter. OCT enables objective evaluation of vitreous reflectivity, with increased signal intensity corresponding to higher particle density and active inflammation. To improve measurement consistency, Keane et al. introduced the VIT/RPE ratio, which normalizes vitreous signal intensity against the retinal pigment epithelium (RPE), serving as an internal reference [29]. This approach reduces acquisition-related variability and allows for more reproducible quantification, independent of lens status or prior vitrectomy [30].

The clinical relevance of this metric was further supported by Sreekantam et al., who demonstrated sensitivity in detecting therapeutic response following sub-Tenon’s triamcinolone acetonide in uveitic cystoid macular edema, with a significant reduction in VIT/RPE-relative intensity from 0.139 to 0.053, correlating with decreased central retinal thickness and improved visual acuity [31]. However, VIT/RPE measurements remain influenced by acquisition parameters such as retinal focus, scan averaging and positioning [32]. In addition, OCT inherently provides localized information, as a single B-scan cannot capture the full spatial distribution of inflammatory changes within the vitreous cavity. To mitigate this limitation, Terheyden et al. proposed acquiring multiple widely spaced OCT scans to improve representativeness [33]. Patient positioning during imaging may also introduce bias, as gravitational settling of inflammatory material toward the inferior vitreous can lead to an underestimation of the inflammatory burden.

Efforts to expand OCT-based evaluation have also targeted previously inaccessible regions of the vitreous. Invernizzi et al. introduced an anterior segment swept-source OCT (AS-SSOCT) approach to visualize the anterior vitreous by adjusting device positioning, enabling imaging of the compartment immediately posterior to the lens [34,35]. Although this technique extends anatomical visualization, assessment remains limited to the anterior vitreous, potentially missing inflammatory changes in the posterior vitreous.

Another promising biomarker has been described by Pichi et al., who identified vitreoretinal interface cells (VRICs) using en face OCT angiography (SS-OCTA) [5]. These cells are thought to participate in immune processes, including antigen presentation and phagocytosis. Increased number and size of these elements have been observed in active uveitis, correlating with disease activity and suggesting their potential as inflammatory biomarkers [36]. However, their localization limits their ability to reflect inflammatory processes in other portions of the vitreous.

Several factors may influence the accuracy and reproducibility of OCT-derived vitreous biomarkers. Media opacities, particularly cataract, may alter signal transmission and affect vitreous reflectivity measurements. In addition, image quality, signal strength, segmentation errors and differences among OCT platforms may introduce measurement variability. These potential confounders should be considered when interpreting quantitative vitreous biomarkers in both clinical practice and research settings [29,35].

Alternative imaging modalities have also been explored. Quantitative ultrasound (QUS) has been used by Mamou et al. to assess vitreous opacities, demonstrating a correlation between increased echo density and reduced contrast sensitivity, as well as decreased patient-reported visual function [37]. However, QUS is not widely available, has yet to be systematically applied to vitritis, and lacks sufficient spatial resolution to detect subtle inflammatory changes.

Ultrasound biomicroscopy (UBM) using high-frequency ultrasound (20–50 MHz) enables high-resolution imaging of the anterior vitreous. Doro et al. demonstrated its utility in detecting snowbanks in intermediate uveitis [38]. Nevertheless, operator dependence, limited posterior segment penetration, and inability to differentiate inflammatory haze from other opacities reduce its value for quantitative assessment [39].

Finally, automated analysis of fundus imaging has been proposed to overcome subjectivity in traditional grading systems. Passaglia et al. developed an image-processing algorithm incorporating high-pass filtering, entropy analysis and power spectrum integration to quantify vitreous haze from color fundus photography. This approach provides a more objective and reproducible evaluation, particularly relevant for clinical trials and research settings requiring standardized outcome measures [40].

### 1.5. Emerging Directions in Vitreous Inflammation Assessment

Emerging innovations offer promising strategies to overcome the limitations of currently available tools for assessing vitreous inflammation.

#### 1.5.1. Integration of Multimodal Imaging Approaches

**Figure 2:**
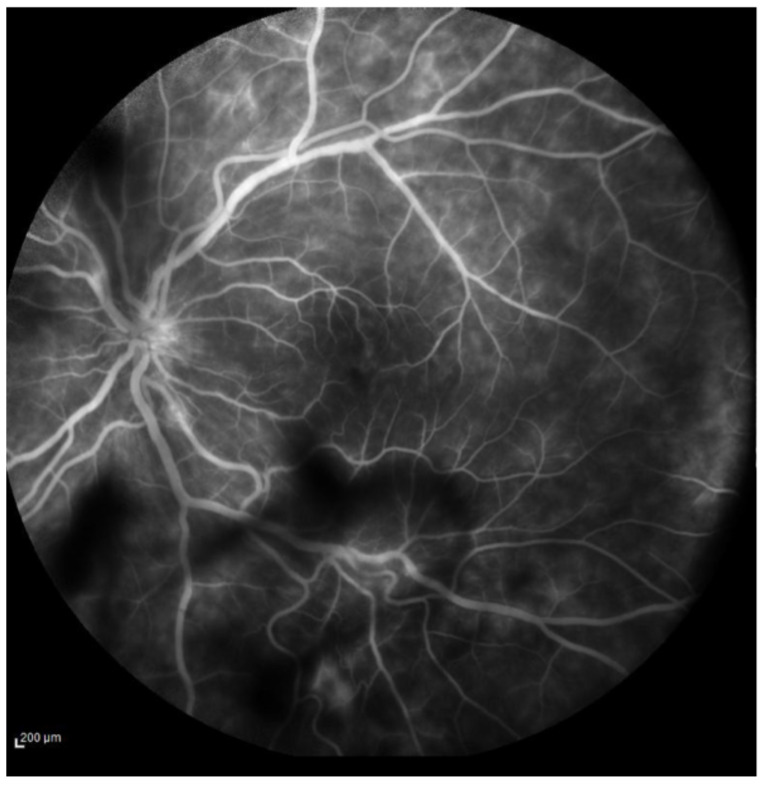
Fluorescein angiography (FA) shows pre-retinal areas of hypofluorescence caused by vitreous inflammatory opacities, resulting in masking of the underlying retinal fluorescence, and vascular leakage due to vasculitis. This example highlights the complementary role of angiographic and vitreous assessment in the evaluation of ocular inflammation. Image obtained from the authors’ institutional database after written informed consent.

Individual imaging modalities, such as OCT, AS-SSOCT, and QUS, provide complementary information but remain limited in their ability to provide a comprehensive and refined evaluation of vitreous inflammation. These limitations highlight the potential of multimodal imaging strategies, in which integrating different techniques may enable a more complete characterisation of inflammatory changes, thereby improving both diagnostic accuracy and treatment monitoring. In animal models, Bradley et al. demonstrated the value of combining OCT, fundus photography and angiographic imaging for quantitative assessment of ocular inflammation [41]. Fluorescein angiography (FA), particularly ultra-widefield FA, remains essential for detecting retinal vasculitis, vascular leakage, and peripheral ischemic changes that may reflect ongoing inflammatory activity despite apparent improvement in vitreous biomarkers [42] (Figure 2). Indocyanine green angiography (ICGA) provides complementary information regarding choroidal inflammation [43]. Fundus autofluorescence (FAF) contributes additional information regarding retinal pigment epithelium dysfunction and cumulative inflammatory damage [44]. Therefore, vitreous biomarkers should be interpreted as part of a multimodal imaging strategy rather than as standalone indicators of disease activity. In this context, magnetic resonance imaging (MRI) may offer additional quantitative information on vitreous inflammation. Oztuerk et al. demonstrated that contrast-enhanced fluid-attenuated inversion recovery with fat suppression (CE-FLAIR-FS) MRI can detect subtle vitreous abnormalities in pediatric uveitis more effectively than conventional contrast-enhanced T1-weighted imaging (CE-T1WI) [45]. These findings suggest that CE-FLAIR-FS MRI may serve as a complementary modality for objective assessment and monitoring of vitreous inflammation.

#### 1.5.2. Advances in Artificial Intelligence and Machine Learning

The integration of artificial intelligence (AI) and machine learning (ML) represents one of the most promising developments in the assessment of vitreous inflammation. These approaches enable improved image segmentation, automated detection of inflammatory features such as cells and flare, and reduced operator-dependent variability. AI-based models trained on large datasets may provide more accurate and standardized evaluation compared to traditional grading systems. Recent studies have demonstrated significant progress in this field. Automated algorithms applied to macular OCT images enable objective grading of vitreous haze, addressing the limitations of subjective clinical assessment [40,46]. Deep learning approaches have further improved sensitivity in detecting subtle changes in vitreous reflectivity. Haggag et al. developed a computer-aided diagnosis (CAD) system combining U-net CNN segmentation with a fully convolutional neural network, achieving 86% accuracy compared to expert grading and outperforming SVM-based methods (70.1%) [47]. Similarly, Mhibik et al. developed a deep learning model based on TensorFlow and DenseNet121 for the automated detection and grading of vitritis on UWF images, achieving 91% sensitivity, 89% specificity, and an AUC of 0.97. While classification across all SUN grades showed limited accuracy (0.61), grouping into broader categories improved performance to 0.75 [48]. In addition, AI-based systems may enable longitudinal analysis of disease progression, supporting personalized therapeutic decision-making.

Despite these promising results, several limitations currently hinder the clinical implementation of AI-based approaches for vitreous inflammation assessment. Most published models have been developed using relatively small and highly selected datasets, often without external validation, limiting their generalizability across different patient populations and imaging platforms. Furthermore, the lack of publicly available datasets and standardized evaluation protocols reduces reproducibility and makes direct comparison between algorithms difficult. Additional challenges include the limited transparency of complex deep learning models, potential algorithmic bias related to training datasets, and unresolved regulatory and ethical considerations regarding data privacy, validation, deployment, and clinical monitoring. Therefore, larger multicenter studies with standardized datasets and external validation are required before AI-based tools can be routinely integrated into clinical practice [46].

#### 1.5.3. Functional Imaging and Molecular Biomarkers

Functional imaging and molecular biomarkers represent an additional avenue for advancing the assessment of vitreous inflammation, providing insight beyond structural changes. Imaging techniques such as MRI with targeted contrast agents may allow for the visualization of inflammatory cell activity, while molecular approaches enable the characterization of cytokine networks, protein expression profiles, and metabolic alterations associated with intraocular inflammation. Cytokine profiling has emerged as a valuable tool for differentiating inflammatory and neoplastic vitreoretinal disorders. Microfluidic immunoassay platforms, such as the sandwich immunofluorescence assay (sIFA) described by Green et al., demonstrated greater sensitivity than conventional ELISA, allowing for cytokine detection in the single-digit femtomolar range and improving the distinction between uveitis and primary vitreoretinal lymphoma [49]. Furthermore, vitreous cytokine analysis has identified biomarkers potentially associated with disease activity and prognosis. Recent observations in vitreoretinal lymphoma reported the first detection of vitreous IL-16, suggesting that longitudinal monitoring of IL-16, together with established biomarkers such as IL-10 and IL-6, may provide additional information regarding the intraocular tumor microenvironment and therapeutic response, although larger validation studies are required [50].

Proteomic and metabolomic analyses of vitreous samples represent additional emerging approaches for characterizing intraocular inflammation. By identifying proteins, signaling pathways, and metabolic signatures associated with inflammatory activity, these techniques may facilitate biomarker discovery, improve disease stratification, and contribute to a more personalized assessment of vitreous inflammation [51,52].

Collectively, molecular characterization of vitreous fluid may complement structural imaging by providing direct biological information regarding intraocular inflammatory processes and disease activity.

#### 1.5.4. Automated Quantification and Standardization

Despite recent advances, further development of standardized quantitative methods remains essential. Current approaches, including the VIT/RPE ratio and manual counting of hyperreflective foci, provide valuable information but remain susceptible to variability. The implementation of fully automated systems capable of quantifying both cellular infiltration and overall inflammatory burden may significantly improve reproducibility and clinical applicability. Future developments may include 3D volumetric imaging, which will allow for the assessment of the spatial distribution of inflammatory cells throughout the vitreous cavity, thereby overcoming the limitations of two-dimensional analysis. In addition, advanced algorithms integrating imaging features with molecular data may enable improved discrimination between inflammatory cells and other vitreous opacities, such as hemorrhage, debris, or degenerative changes, enhancing diagnostic specificity.

## 2. Conclusions

Accurate assessment of vitreous inflammation remains a cornerstone of uveitis management. Traditional clinical grading systems, although widely used, are limited by subjectivity and interobserver variability, reducing their reliability for monitoring subtle changes and guiding therapeutic decisions.

Recent advances in imaging technologies, particularly OCT-based metrics and automated quantification of vitreous reflectivity and cellular aggregates, represent a significant step toward objective evaluation of inflammatory burden. Nevertheless, no single modality currently provides a comprehensive assessment of the entire vitreous cavity, highlighting the need for integrated diagnostic approaches.

Future developments will likely rely on a combination of multimodal imaging, artificial intelligence-driven analysis and emerging molecular biomarkers to achieve standardized and reproducible quantification of vitreous inflammation. These advances may enable more accurate disease monitoring, improved differential diagnosis, and personalized therapeutic strategies.

Ultimately, the transition from subjective clinical grading to objective quantification of inflammatory burden represents a critical step toward precision medicine in ocular inflammatory diseases. Establishing reliable and reproducible biomarkers will be essential not only for routine clinical practice but also for developing robust endpoints in clinical trials and advancing uveitis research.

## Data Availability

No new data were created or analyzed in this study.
